# Developmental Expression of Kv Potassium Channels at the Axon Initial Segment of Cultured Hippocampal Neurons

**DOI:** 10.1371/journal.pone.0048557

**Published:** 2012-10-31

**Authors:** Diana Sánchez-Ponce, Javier DeFelipe, Juan José Garrido, Alberto Muñoz

**Affiliations:** 1 Department of Functional and Systems Neurobiology, Instituto Cajal, Consejo Superior de Investigaciones Científicas, Madrid, Spain; 2 Department of Molecular, Cellular and Developmental Neurobiology, Instituto Cajal, Consejo Superior de Investigaciones Científicas, Madrid, Spain; 3 Laboratorio Cajal de Circuitos Corticales, Centro de Tecnología Biomédica, Universidad Politécnica de Madrid, Madrid, Spain; 4 Department of Cell Biology, Complutense University, Madrid, Spain; 5 Centro de Investigación Biomédica en Red de Enfermedades Neurodegenerativas (CIBERNED), Madrid, Spain; University of Houston, United States of America

## Abstract

Axonal outgrowth and the formation of the axon initial segment (AIS) are early events in the acquisition of neuronal polarity. The AIS is characterized by a high concentration of voltage-dependent sodium and potassium channels. However, the specific ion channel subunits present and their precise localization in this axonal subdomain vary both during development and among the types of neurons, probably determining their firing characteristics in response to stimulation. Here, we characterize the developmental expression of different subfamilies of voltage-gated potassium channels in the AISs of cultured mouse hippocampal neurons, including subunits Kv1.2, Kv2.2 and Kv7.2. In contrast to the early appearance of voltage-gated sodium channels and the Kv7.2 subunit at the AIS, Kv1.2 and Kv2.2 subunits were tethered at the AIS only after 10 days *in vitro*. Interestingly, we observed different patterns of Kv1.2 and Kv2.2 subunit expression, with each confined to distinct neuronal populations. The accumulation of Kv1.2 and Kv2.2 subunits at the AIS was dependent on ankyrin G tethering, it was not affected by disruption of the actin cytoskeleton and it was resistant to detergent extraction, as described previously for other AIS proteins. This distribution of potassium channels in the AIS further emphasizes the heterogeneity of this structure in different neuronal populations, as proposed previously, and suggests corresponding differences in action potential regulation.

## Introduction

The non-uniform distribution of specific of voltage-gated K^+^ (Kv) channels and their restriction to discrete neuronal domains is thought to contribute to the control of neuronal excitability. Indeed, these channels are believed to influence different properties of neurons, including resting membrane potential, waveform shape, action potential (AP) firing pattern, transmitter release and synaptic strength. The importance of Kv channels in neuronal function is reflected by the neurological alterations induced by mutations or diseases that disrupt K^+^ channel expression, including episodic ataxia and epilepsies [1], [2], [3], [4], [5], [6]. In the plasma membrane, Kv channels are actually complexes made up of four voltage-sensing and pore-forming subunits, each generated from a family of over 35 subunits divided into 12 subfamilies (Kv1–12) [7], [8]. These complexes may assemble with auxiliary β subunits what may influence the expression, localization and biophysical properties of Kv channels [9], [10], [11]. The axon initial segment (AIS) is a neuronal domain that is densely populated by voltage-gated ion channels, and it is a structure that is critical for input integration and action potential generation [12], [13], [14], [15]. In addition to voltage-gated Na^+^ (VGSC) [16], [17], [18], [19], [20] and Ca^2+^ (Ca_v_) channels [12], [21], [22], the AIS of neocortical and hippocampal principal cells is characterized by the expression of Kv1, Kv2 and Kv7 channels [17], [18], [19], [20], [23], [24], [25], [26]. The distinct subunit composition of these channels confers distinct biophysical properties to the ion currents mediated by each of them. Together with heterogeneous expression and localization of such channels in the AIS, they are likely to contribute to the electrophysiological variability between neuronal populations, and the corresponding differences in AP initiation and/or propagation [17], [18], [19], [20], [27], [28], [29], [30], [31].

**Figure 1:**
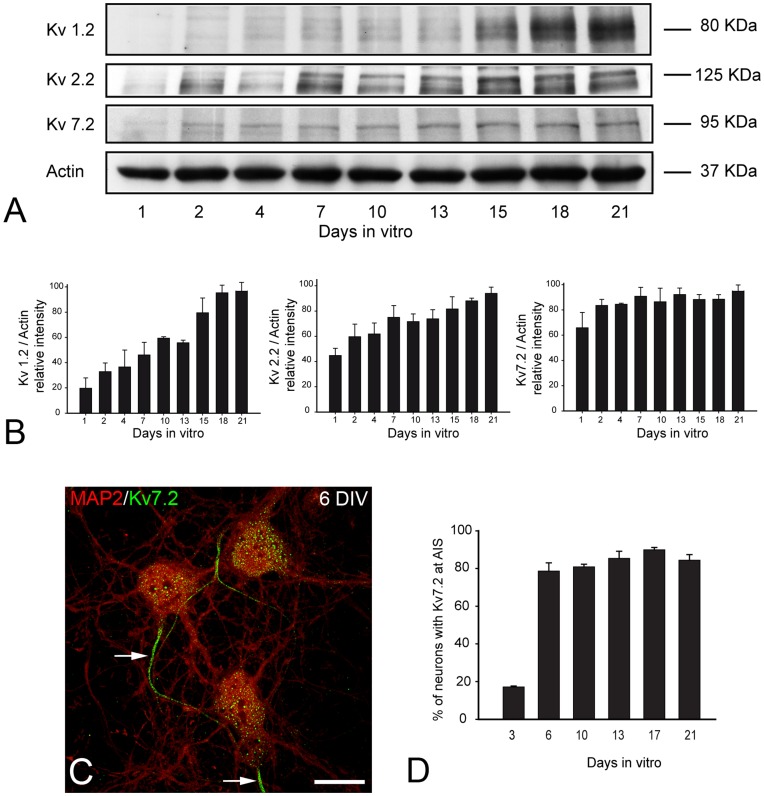
Developmental expression of potassium channels in cultured hippocampal neurons. (A) Western blot of Kv1.2, Kv2.2 and Kv7.2 in hippocampal neurons cultured at high density (50,000/cm^2^) for different intervals (from 1 to 21 DIV) in control conditions. (B) Histograms show Kv1.2, Kv2.2 and Kv7.2 expression normalized to actin when quantified densitometry of Western blots. The data represent the mean ± SE of three independent experiments. Note the delayed onset of Kv1.2 expression as compared with that of Kv2.2 and Kv7.2. (C) Photomicrograph of hippocampal neurons cultured for 6 DIV and double immunostained for Kv7.2 (green) and MAP2 (red). Note the early expression of Kv7.2 in a single process emerging from the cell body (arrows). (D) Histogram shows the percentage (mean ± SE) of neurons expressing Kv7.2 at the AIS at different developmental stages *in vitro*. Scale bar = 16 µm.

Kv1 channels are characterized by low thresholds, as well as rapid activation and slow inactivation kinetics, and they are known to modulate the threshold, initiation, shape and propagation rate of APs, as well as neurotransmitter release and synaptic efficacy [25], [32], [33], [34], [35], [36]. Kv2 or delayed rectifier Kv channels regulate somatodendritic excitability and Ca^2+^ influx in hippocampal and cortical neurons during periods of repetitive high frequency firing [37], [38], [39], [40], [41], [42], [43]. These channels are also localized at the AISs of different neuronal populations, where they contribute to the maintenance of AP amplitude by regulating the inter-spike potential during high frequency firing [44], [45]. Kv7 channels are localized at the AIS [23], [46], [47], [48], [49], [50], [51] and they underlie the M-current. These channels regulate resting potential and AP firing and they are characterized by low-threshold, slow activation gating at negative voltages, and sustained activity and non-inactivation near the AP threshold [47], [52], [53], [54].

Despite the functional importance of the AIS, the timing and the intracellular mechanisms involved in the compartmentalization of Kv channels at the AIS remain poorly understood. A key aim of the present study was to describe the distribution, developmental expression and co-localization of different Kv channel types in the AIS of cultured hippocampal neurons, a model commonly used to study the development of neuronal polarity and axonal maturation [55]. In cultured hippocampal neurons, ankyrin G is one of the earliest markers to be detected at the AIS and it is essential for the tethering of other proteins that appear early during AIS development, such as VGSC [56], [57], [58]. Like VGSCs, Kv7 channels (Kv7.2 and Kv7.3 subunits) contain a common ankyrin G binding domain that is required for their targeting to the AIS. Together with the adhesion molecules NrCAM and Neurofascin 186, accumulation of Kv7 channels at the AIS is critically dependent on the interaction of ankyrin G with the actin cytoskeleton via βIV spectrin [16], [46], [48], [49], [56], [57], [59], [60], [61], [62], [63]. Correct tethering of these proteins also depends on the structural integrity of actin and on the microtubule cytoskeleton in the AIS [64], [65], [66], [67]. Further AIS maturation in cultured hippocampal neurons involves the expression of GABA_A_ receptor subunits and gephyrin clusters [68], as well as the acquisition of the cisternal organelle. The latter structure is involved in Ca^2+^ regulation and reaches neurochemical maturation during the second week *in vitro* [69], [70]. To date, the precise temporal distribution of Kv1 and Kv2 channels in the AIS, along with the associated trafficking and clustering mechanisms, has not been fully elucidated [19], [20], [24]. Therefore, we have studied the temporal and spatial distribution of Kv1.2 and Kv2.2 subunits during AIS development, and the role of the submembranous actin cytoskeleton in this process. Our results show that Kv1.2 and Kv2.2 expression are mutually exclusive in the AIS of cultured hippocampal neurons, and that their localization in this region is dependent on ankyrin G, yet independent of actin cytoskeleton integrity.

**Figure 2:**
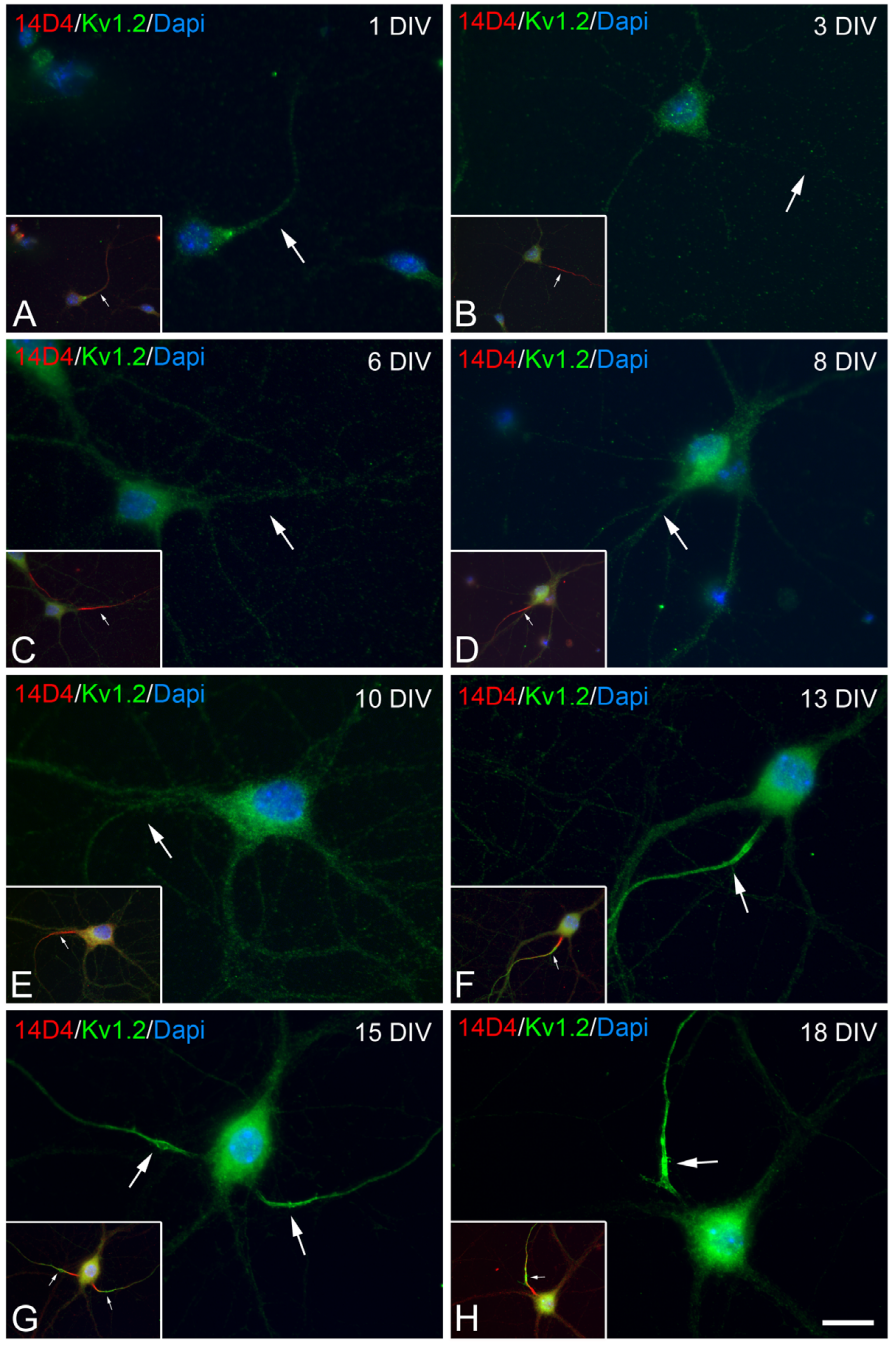
Kv1.2 is concentrated at the AIS during axonal maturation *in vitro*. Hippocampal neurons were grown for 1, 3, 6, 8, 10, 13, 15 and 18 days at low density (5,000/cm^2^), fixed in 4% PFA, and doubled stained with antibodies against Kv1.2 (green) and 14D4 antibodies (red) to identify the AIS. Note that 14D4 staining is detected at the moment of axon outgrowth in the nascent axon (A, B) and it is restricted to the AIS as the axon elongates (arrows). Confocal microscopy photomicrographs showing Kv1.2 immunostaining in hippocampal neurons cultured for up to 10 DIV (A–E). Staining is light and localized to the soma and neurites. After 10 DIV (F–H), intense Kv1.2 immunostaining is observed in the distal AIS. See Figure 3 for quantification. Scale bar = 18 µm.

## Materials and Methods

### Neuronal Cultures

Hippocampal neurons were obtained from E17 mouse embryos and prepared as described previously [55]. Mice were obtained from the Cajal Institute animal facility. Pregnant female mice and embryos were sacrificed by cervical dislocation and decapitation respectively following the guidelines of Council of Europe Convention ETS123, recently revised as indicated in the Directive 86/609/EEC. In addition all protocols were approved by the institutional animal care and use committee (Subcomité de Bioética, CSIC; Institutional review board; IRB 0007851).

**Figure 3:**
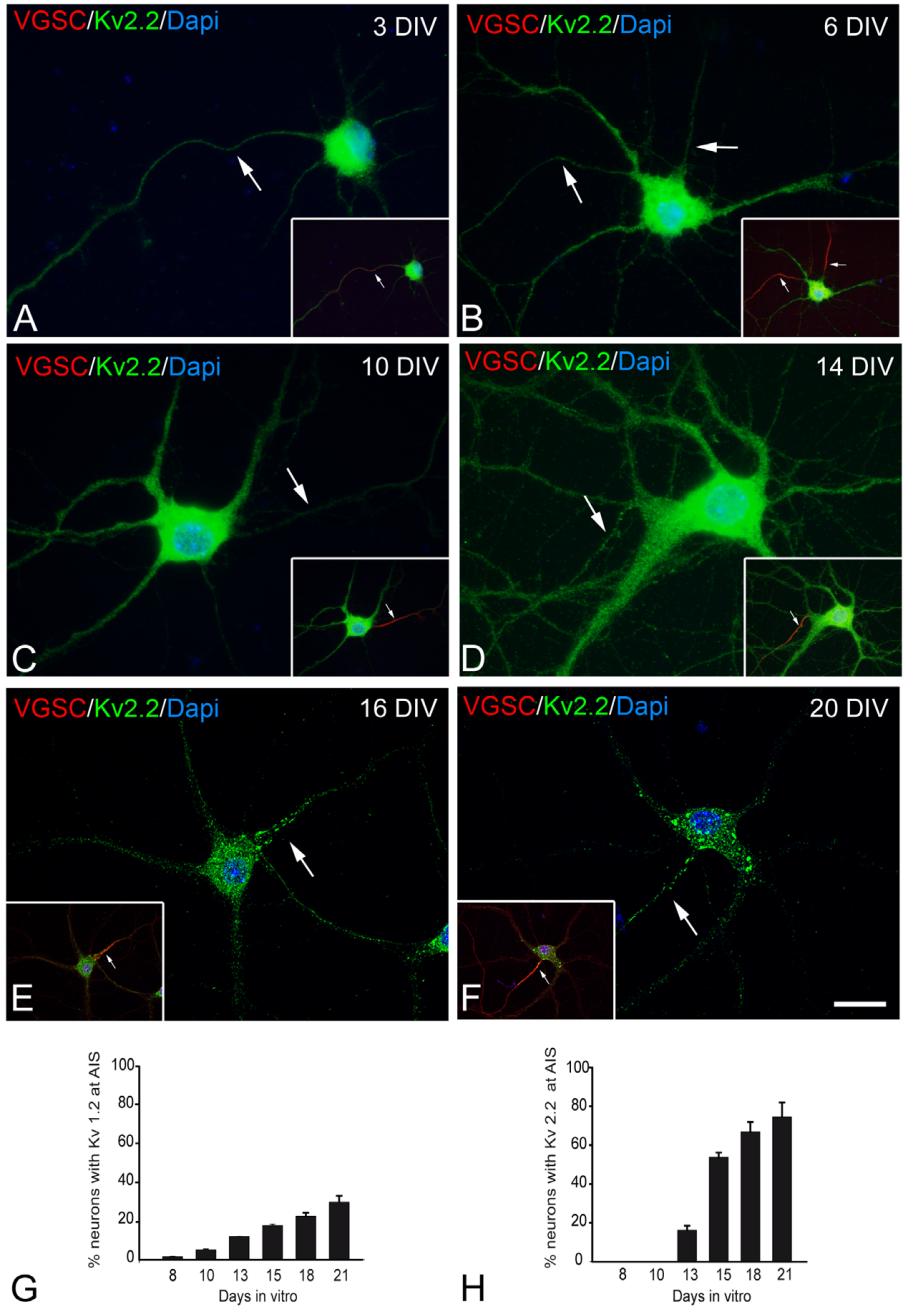
Kv2.2 concentration at the AIS increases during axonal maturation *in vitro*. Confocal microscopy photomicrographs showing representative hippocampal neurons cultured for 1, 3, 6, 8, 10, 13, 15, 18 and 20 days at low density (5,000/cm^2^), fixed in 4% PFA, and stained with antibodies against Kv2.2 (green) and VGSC (red). According to a previous study (Sánchez Ponce et al., 2008), VGSCs concentrate at the AIS (arrows) after 3 DIV. Note that moderate Kv2.2 immunostaining is localized homogeneously in the soma and proximal processes at all developmental stages in culture (A–C). After 14 DIV (D–F), Kv2.2 expression is evident in the axon in patches mainly distributed in the proximal region of the AIS. Histogram shows the percentage of neurons expressing Kv1.2 (G) and Kv2.2 (H) at the AIS at different developmental stages *in vitro* (the data represent the mean ± SE from three independent experiments). Scale bar = 18 µm (A–D) and 16 µm (E–F).

Briefly, after dissection of the hippocampus, tissue pieces were washed three times in Ca^2+^/Mg^2+^-free HSBB and digested for 15 min in the same solution containing 0.2% trypsin. The tissue was washed three times in Ca^2+^/Mg^2+^-free HBSS and dissociated with a fire-polished Pasteur pipette. The cells were counted, resuspended in plating medium (MEM with 10% Horse Serum and 0.6% glucose) and plated on polylysine coated coverslips (1 mg/ml) at a density of 5,000 cells per cm^2^ (low density) for immunostaining, or 50,000 cells per cm^2^ (high density) for Western blots. After 2 hours, the medium was replaced with neuronal culture medium (Neurobasal medium supplemented with B-27 and glutamax-I). To maintain the neurons for 21 days *in vitro* (DIV), the cells were transferred to 60 mm plates containing astrocyte monolayers that had been cultured in neuronal culture medium for 24 h previously. 1-β-D-arabinofuranosylcytosine (AraC: 5 µM) was added to the culture after 3 days to prevent astroglial cell growth, and in some cases neurons were treated between 15 and 17 DIV with 5 µM cytochalasin D (Sigma) to impede actin polymerization. For detergent extraction, neurons were maintained in culture for 21 DIV, washed briefly in PBS and then treated for 15 minutes at 37°C with 1% Triton X-100 in cytoskeletal buffer (2 mM MgCl_2_, 10 mM EGTA, 60 mM Pipes [pH 7.0]), as described previously [64]. For nucleofection experiments, the plasmids were introduced into hippocampal neurons by nucleofection prior to plating (Amaxa Bioscience, Koln, Germany), according to the manufacturer’s instructions. Nucleofection was performed using 3 µg of total DNA and the plasmids used for transient expression were: scrambled negative control shRNA in a pGFP-V-RS plasmid and a shRNA-AnkG (sequence: TCGGATAGGTCCTACACCTTGAACAGAAG) in a pGFP-V-RS plasmid (Origene, Rockville, MD, USA). The effects of nucleofection were analyzed at 18 DIV.

**Figure 4:**
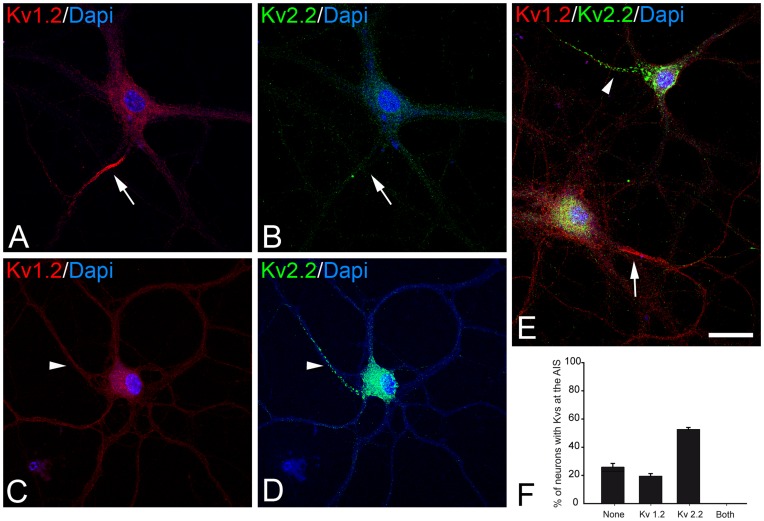
Lack of Kv1.2 and Kv2.2 colocalization at the AIS of cultured hippocampal neurons. A–B and C–D: Pairs of representative confocal microscopy photomicrographs of hippocampal neurons cultured for 18 days, double immunostained for Kv1.2 (red) and Kv 2.2 (green), and counterstained with DAPI (A–D). Note that Kv1.2-expressing AISs (arrows) lack Kv2.2 immunostaining (Kv2.2) and vice versa (E). Histogram shows the proportion of neurons expressing Kv1.2, Kv2.2, neither or both at the same AIS at 18 DIV (the data represent the mean ± SE from three independent experiments). Scale bar = 25 µm.

### Western Blotting

Protein samples were prepared from hippocampal neurons cultured at high density (50,000/cm^2^) in control conditions. After different times in culture (from 1 DIV to 21 DIV), the plates were washed twice with cold PBS, the neurons were lysed and then homogenized in a buffer containing: 20 mM HEPES [pH 7.4]; 100 mM NaCl; 100 mM NaF; 1% Triton X-100; 1 mM sodium orthovanadate; 10 mM EDTA; and a complete protease inhibitor cocktail (Roche Diagnostics, Mannheim, Germany). The lysates were boiled for 10 minutes, separated by SDS-PAGE on 8% acrylamide gels and transferred to nitrocellulose membranes. The membranes were incubated overnight at 4°C with primary antibodies in blocking solution (PBS, 0.2% Tween-20 and 5% BSA): mouse anti-Kv1.2 (1∶1,000; Neuromab, UC Davis, USA); rabbit anti-Kv2.2 (1∶500; Alomone, Jerusalem, Israel); mouse anti-Kv7.2 (KCNQ2, 1∶500; Neuromab) and mouse anti-β-actin (1∶5,000; Sigma, St Louis MO, USA). After washing, the membranes were incubated with the corresponding peroxidase conjugated secondary antibody for 2 h at room temperature, and antibody binding was visualized by ECL (Amersham). Densitometry was performed using an imaging densitometer (GS-800, BioRad). Background level was subtracted using a whole-image background subtraction tool (Quantity One software, BioRad).

**Figure 5:**
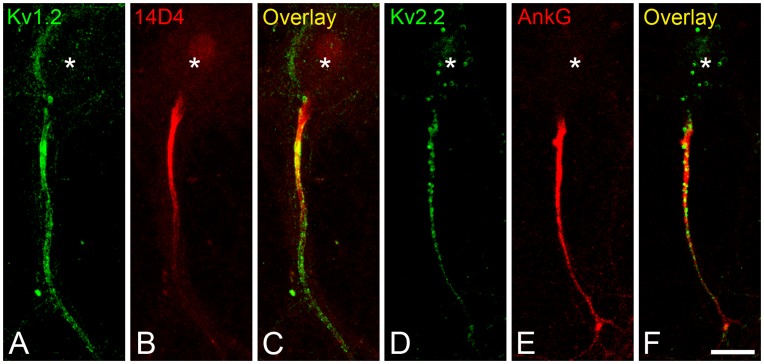
AIS resistance to detergent extraction. Hippocampal neurons cultured for 21 DIV were incubated for 15 min in a buffer containing 0.5% Triton X-100 before fixation (see Experimental methods) and analyzed by confocal microscopy. After detergent extraction, Kv1.2 (A) and Kv2.2 (D) were still present in the AIS, and they colocalized with the detergent resistant AIS markers 14D4 (B) and ankyrin G (E), respectively. Asterisks indicate the location of the neuronal soma. Scale bar = 12 µm.

### Immunocytochemistry

After different times in culture, neurons were fixed in 4% paraformaldehyde for 20 minutes and then washed in PBS. For immunostaining, the coverslips were treated with 50 mM NH_4_Cl and incubated in blocking buffer for 45 minutes (PBS, 0.22% gelatin and 0.1% Triton X-100). After blocking non-specific binding, the coverslips were incubated for 1 h at room temperature with the primary antibodies to Kv channel subunits diluted in blocking buffer: rabbit anti-Kv2.2 (1∶250, Alomone); mouse anti-Kv1.2 (1∶250; Neuromab); mouse anti-Kv 7.2 (KCNQ2, 1∶200; Neuromab). To identify the AIS we used mouse anti-Pan sodium channels (1∶100; Sigma) and mouse anti-ankyrin G (1∶200; Neuromab) antibodies and rabbit antibodies (14D4) raised against phospho (p-32)-IκBα (1∶500; Cell Signalling, Beverly, MA, USA) that recognize an uncharacterized phosphorilated protein present at the AIS [71]. Mouse anti-Tau-1 (1∶1,000; Sigma) and chicken anti-MAP2 (1∶5,000; Abcam, Cambridge, UK) antibodies were used to reveal axonal and neuronal morphology respectively. In some neurons, actin filaments were also stained with Alexa 488 phalloidin (1∶100; Invitrogen, A-12379). The secondary antibodies used were donkey anti-mouse, anti-rabbit or anti-chicken cupled to Alexa 488, Alexa 594 or Alexa 647. After staining coverslips were counterstained with DAPI (1∶1000, Calbiochem, San Diego, CA, USA) and mounted in Fluoromount G (Southern Biotech, Birmingham, AL, USA). Images were obtained using a DP70 camera attached to an Olympus BX51 fluorescence microscope, or by laser scanning confocal microscopy (Zeiss 710). Z sections were recorded at 0.2–1-µm intervals through separate channels and ZEN 2009 software (Zeiss) was used to construct composite images from each optical series by combining the images recorded through the different channels. In all cases, Adobe Photoshop CS4 software was used to generate the figures (Adobe Systems Inc., San Jose, CA, USA). The cell counts in the different experimental conditions were compared by the unpaired t-test using Sigma Plot 11.0 software.

**Figure 6:**
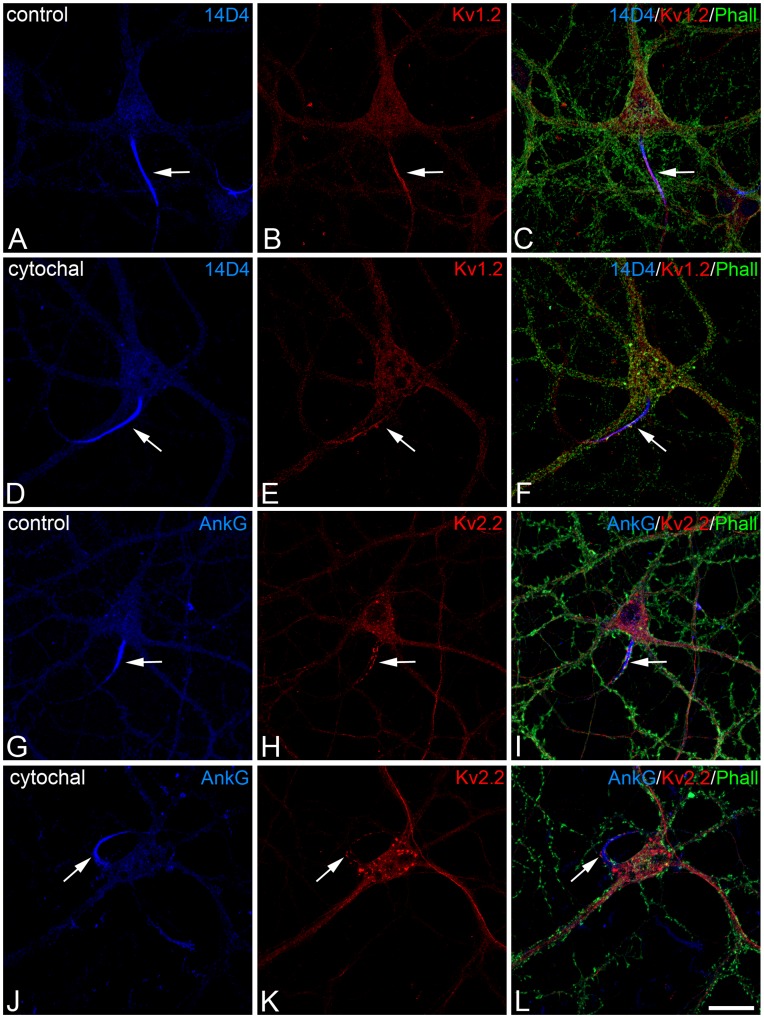
AIS Kv channel expression is not dependent on the actin cytoskeleton. Confocal microscopy photomicrographs show that Kv1.2 (A–F) and Kv2.2 (G–L) accumulation in the AIS is not affected by cytochalasin D. Hippocampal neurons were exposed to DMSO (control, A–C, G–I) or cytochalsin D (5 µM; D–F, J–L) from 15 to 17 DIV, double stained for 14D4 or ankyrin G (blue) and Kv1.2 (red, A–F) or Kv2.2 (red, G–L), and stained with Alexa 488 phalloidin to reveal F-actin. Note the presence of Kv1.2 and Kv2.2 at the AIS in both control and cytochalasin D-treated neurons. Scale bar = 25 µm (A–F) and 30 µm (G–L).

## Results

### Voltage-gated Potassium Channel Expression in the Developing AIS of Cultured Hippocampal Neurons

We first analyzed the expression of voltage-gated potassium channels in hippocampal neurons cultured at high density for different intervals up to three weeks. The total expression of the different Kv subunits was analyzed in Western blot and the results were normalized to the expression of β-actin (Fig. 1). Kv1.2 subunit expression was only weakly detected during the first days *in vitro*, yet it increased progressively from about 13 DIV (Fig. 1A–B). By contrast, Kv2.2 subunit expression was clearly evident from the first day in culture and it increased progressively thereafter. The pattern of Kv7.2 subunit expression closely resembled that observed for Kv2.2, consistent with previous studies describing the early onset of Kv7.2 expression at the AIS. This Kv7.2 expression is dependent on its binding to ankyrin G [48], which is expressed in the developing AIS from 3 DIV [58].

We next used immunocytochemical analysis to study the localization of these voltage-gated potassium channels in the developing AIS of cultured hippocampal neurons. When we studied the localization of Kv7.2 subunit in our cultures (Fig. 1C–D), it was already present in the AIS of 17.15 (±0.49%) of neurons after 3 DIV and from 6 DIV onwards, it was detected in ∼80% of neurons: 78.64 (±4.38%) at 6 DIV; 80.88 (±1.39%) at 10 DIV; 85.39 (±3.76%) at 15 DIV; 89.96 (±1.19%) at 17 DIV; and 84.44 (±2.96%) at 21 DIV. Kv7.2 was distributed homogeneously in the AIS and it was detected along the distal and proximal regions at all stages of development (Fig. 1C). As the expression patterns of Kv7.2 and Kv7.3 at the AIS along with the mechanisms that allow their concentration at the AIS have been well characterized [48], [50] we subsequently focused on Kv1 and Kv2 channel expression. Accordingly, the results described below demonstrate that the trafficking of Kv1 and Kv2 channels towards the axon lags behind that of sodium channels and Kv7.2 channels.

**Figure 7:**
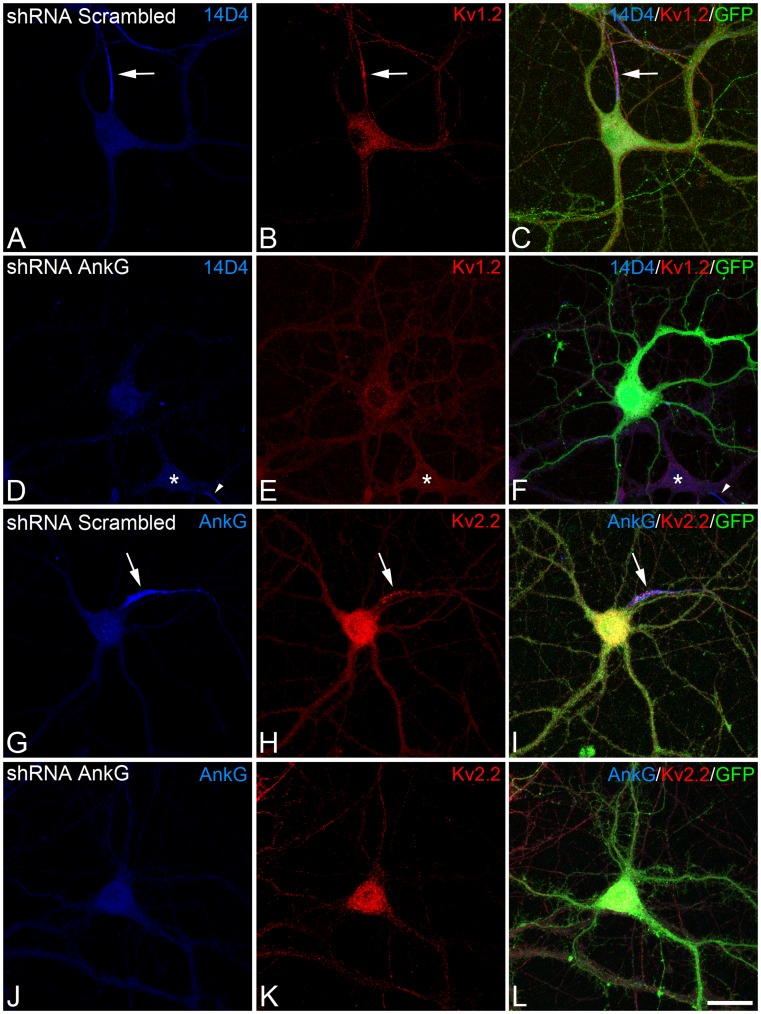
AIS Kv channel concentration is dependent on ankyrin G. Confocal microscopy photomicrographs show that interference RNAs against ankyrin G impair the concentration of Kv1.2 and Kv2.2 at the AIS. Before plating, hippocampal neurons were nucleofected with plasmids expressing scrambled shRNA (A–C, G–I) or ankyrin G shRNA (D–F, J–L), and subsequently cultured until 18 DIV. The neurons were then double stained with antibodies against ankyrin G or pIκBα (14D4 blue) and Kv1.2 or Kv2.2 (red). Nucleofected neurons were identified by GFP fluorescence. Ankyrin G, the protein recognized by 14D4 antibodies, Kv1.2 and Kv2.2 concentrated in the AIS of neurons expressing scrambled shRNA plasmids (A and G) and in non-nucleofected neurons (arrowhead in D–F). However, in the absence of ankyrin G in neurons nucleofected with ankyrin G shRNA (D–F, J–L), no Kv1.2 or Kv2.2 immunostaining was observed at the AIS. Note that patches of Kv2.2 immunostaining in the cell somata were not affected by ankyrin G interference (K). Scale bar = 25 µm. Arrows indicate AISs and asterisks indicate the somas of non-nucleofected neurons.

### Kv1.2 Expression in the AIS of Cultured Hippocampal Neurons

To study the expression of low threshold voltage-gated potassium (Kv1) channels during the development of neuronal polarity and AIS maturation, hippocampal cells were cultured for different intervals and stained with antibodies raised against the Kv1.2 subunit (Fig. 1). Kv1.2 was expressed in the nascent axon after 36 h in culture, as identified by 14D4 staining, which co-localizes with the axonal marker SMI-31 [58]. In parallel with axon elongation, there was a progressive concentration of 14D4 immunostaining in the AIS, whereas diffuse Kv1.2 staining was observed in neurons, predominantly in the soma. Indeed, Kv1.2 did not concentrate in the AIS until around 8 DIV (Fig. 2 A–E). Consistent with the findings in Western blots, Kv1.2 immunostaining was more intense and specifically concentrated to the AIS of some neurons after 10 DIV (Fig. 2F–H). Indeed, the percentage of neurons exhibiting Kv1.2 staining restricted to the AIS (Fig. 3G) increased after 8 DIV to ∼30% of cultured neurons by 21 DIV (n = 3): 1.46 (±0.25%) at 8 DIV; 5.15 (±0.39%) at 10 DIV; 12.1 (±0.07%) at 13 DIV; 17.67 (±0.63%) at 15 DIV; 22.56 (±1.76%) at 17 DIV; and 29.75 (±1.41%) at 21 DIV. Kv1.2 immunostaining was homogeneous, unclustered and mainly concentrated in the distal region of the AIS, while the proximal region in which 14D4 staining was detected, exhibited no Kv1.2 expression at any developmental stage (Fig. 2F–H).

### Kv2.2 Expression at the AIS of Cultured Hippocampal Neurons

To study the expression of delayed rectifier voltage-gated potassium channel (Kv2) during the development of the AIS, hippocampal cells cultured for different intervals were stained with antibodies directed against the Kv2.2 subunit (Fig. 3), and for sodium channels to identify the AIS. In Western blots, Kv2.2 immunostaining was evident from the initial stages of neuronal differentiation. Indeed, immunocytochemistry revealed diffuse staining in the soma and proximal processes at all times in culture. In addition, after 14 DIV (Fig. 3D–F) there were intense patches of Kv2.2 immunostaining in the AIS, mainly in the proximal region, in contrast to the pattern of Kv1.2 expression observed. The proportion of neurons exhibiting clustered and polarized Kv2.2 expression (Fig. 3H) increased progressively after 14 DIV, representing over 70% of the cultured neurons at 21 DIV (n = 3): 16.32 (±2.19%) at 10 DIV; 53.76 (±2.35%) at 14 DIV; 66.89 (±5.02%) at 17 DIV and 74.57 (±7.36%) at 21 DIV.

### Mutually Exclusive Kv1.2 and Kv2.2 Expression in the AIS

The differential localization of low-threshold Kv1.2 (distal) and delayed rectifier Kv2.2 potassium channels (proximal) in the AIS suggests that each potassium channel is expressed in a specific region of this structure. Indeed, when we double-stained 21 DIV neurons using antibodies against Kv1.2 and Kv2.2 (Fig. 4), the expression of these subunits at the AIS was mutually exclusive and they were localized in distinct neuronal populations (Fig. 4). Kv1.2 subunits were detected at the AIS of 19.47 (±1.85%) of neurons, which in turn exhibited no Kv2.2 immunostaining in the AIS (arrows in Fig. 4). By contrast, in neurons with clustered Kv2.2 expression in the soma and AIS (52.67±1.34%; arrowheads in Fig. 4) no Kv1.2 immunostaining was evident in the AIS. In our experimental conditions, we failed to observe neurons exhibiting Kv1.2/Kv2.2 double immunostaining in the AIS. Moreover, a significant percentage of neurons expressed neither Kv1.2 nor Kv2.2 in the AIS (27.85% ±0.63: Fig. 4F).

### Kv1.2 and Kv2.2 Localization at the AIS is Dependent on Ankyrin G and Independent of the Integrity of the Actin Cytoskeleton

To identify the mechanisms underlying the localization of Kv1.2 and Kv2.2 to the AIS, we first evaluated the resistance of Kv1.2 and Kv2.2 to detergent extraction, a property common to other proteins concentrated in the AIS, such as ankyrin G and the protein recognized by 14D4 immunostaining [16], [58], [64], [71], [72]. In 21 DIV hippocampal neurons, Kv1.2 (Fig. 5A) and Kv2.2 (Fig. 5D) expression was still evident in the AIS after extraction with 0.5% TX-100 (15 min at 37°C), as identified by 14D4 and ankyrin G immunostaining, respectively (Fig. 5). This suggests that potassium channels in the AIS associate with the cytoskeleton and/or scaffolding proteins that are resistant to detergent extraction, such as ankyrin G.

We also assessed whether the localization of Kv1.2 and Kv2.2 in the AIS was dependent on the integrity of the actin cytoskeleton, which is necessary to preserve the structure and function of the AIS [13], [64], and to maintain the structure and neurochemical features of the cisternal organelle [70]. Actin microfilaments were disrupted in neurons by exposing them to cytochalasin D (5 µM) from 15 to 17 DIV, as witnessed by the altered patterns of phalloidin staining when compared to control neurons (Fig. 6I and L). However, neither the expression nor the distribution of Kv1.2 or Kv2.2 was altered in the AIS following exposure to cytochalasin D (see arrows in Fig. 6). Hence, the polymerized state of actin microfilaments does not actively influence the distribution and tethering of Kv1.2 or Kv2.2 channels in the AIS.

We next investigated the role of ankyrin G in the retention of Kv1.2 and Kv2.2 potassium channels, nucleofecting neurons with plasmids expressing scrambled or ankyrin G shRNA and GFP, and maintaining them in culture until 18 DIV. The absence of ankyrin G expression in the AIS of neurons nucleofected with ankyrin G shRNA was verified by ankyrin G immunostaining. No ankyrin G expression was detected in any of the processes emanating from the cell soma of the vast majority of GFP-positive ankyrin G shRNA-nucleofected neurons (Fig. 7J). Moreover, another AIS marker, recognized by 14D4 immunostaining [71], was absent from ankyrin G shRNA-nucleofected neurons (Fig. 7D), as described previously [72]. By contrast, ankyrin G expression persisted in the AIS of neurons nucleofected with scrambled shRNA plasmids (Fig. 7G). In parallel with the loss of ankyrin G and 14D4 staining, no tethering of Kv1.2 or Kv2.2 was detected by immunostaining in the AIS of neurons nucleofected with ankyrin G shRNA, or in any other process emanating from the soma (Fig. 7B, E, H and K). However, in control nucleofected neurons (distinguished by GFP staining), Kv1.2 (Fig. 7A–C) and Kv2.2 (Fig. 7G–I) expression remained localized in the AIS, with a similar distribution to that observed in non-nucleofected neurons (Fig. 2 and 3). Data from three independent experiments showed that the percentage of neurons expressing Kv1.2 at the AIS fell significantly (p≤0.001), from 16.23 (±2.65%) in scrambled shRNA-nucleofected neurons (total number of nucleofected neurons = 127) to 1.1 (±1.1%) in shRNA ankyrin G-nucleofected neurons (106 nucleofected neurons. Similarly, the clusters of Kv2.2 immunostaining observed in the AIS disappeared in the absence of ankyrin G, while Kv2.2 expression in the soma was unaffected (Fig. 7 J–L). The mean percentage of neurons expressing Kv2.2 at the AIS also fell (p≤0.001) from 71.83 (±1.88%) in scrambled shRNA nucleofected neurons (111 nucleofected neurons) to 1.89 (±0.97%) in shRNA ankyrin G-nucleofected neurons (128 nucleofected neurons). These results, strongly suggest that Kv1.2 and Kv2.2 tethering and clustering at the AIS is dependent on ankyrin G but not on the actin cytoskeleton.

## Discussion

The present findings indicate that in contrast to the early expression of voltage-gated sodium channels (VGSC) and Kv7 potassium channels in the AIS, Kv1.2 and Kv2.2 subunits are first tethered at the AIS of cultured hippocampal neurons after 10 days *in vitro* (DIV). Furthermore, after 21 DIV Kv1.2 and Kv2.2 are distributed distinctly in the AIS, with each subunit largely restricted to distinct populations of neurons. Our results show that the accumulation of Kv1.2 and Kv2.2 subunits in the AIS is resistant to detergent extraction and like other AIS proteins, it is dependent on the presence of ankyrin G. Moreover, the presence of Kv1.2 and Kv2.2 subunits in the AIS is not affected by the disruption of the actin cytoskeleton.

### AIS Maturation

Cultured hippocampal neurons are widely used as a model to study the development of neuronal polarity [55]. This process begins with the specification of the axon, followed by its subsequent elongation and the development of functionally specialized subdomains, including the AIS. These processes require the synthesis, transport and precise spatial and temporal localization of membrane and cytoskeletal components. Among the first markers detected at the AIS of cultured neurons are ankyrin G [56], [57], the protein recognized by 14D4 immunostaining [58], [71] and casein kinase 2 [72], which concentrate at the AIS of the short nascent axon. Ankyrin G is responsible for the accumulation of other structural and functional proteins to the AIS, including VGSC [16], [56], [57], which concentrate in the AIS shortly after ankyrin G accumulation in this region [57], [58]. We found that the ankyrin G-dependent targeting and accumulation of Kv7 channels (Kv7.2 subunit) in the AIS [48], [49] was a relatively early event in AIS maturation (3–6 DIV), although it occurred after VGSC expression [58]. This contrasts with the late onset of Kv1 and Kv2 channel expression, which begin to concentrate at the AIS during the second week *in vitro* (10 DIV). The late expression of Kv1 and Kv2 channels in the AIS of cultured hippocampal neurons is concomitant with the appearance of gephyrin and GABA_A_ receptor subunits at the AIS [68], and the acquisition of the cisternal organelle, which is involved in Ca^2+^ regulation and reaches neurochemical maturation during the second week *in vitro* [69], [70]. Further studies will be required to determine whether these late events in AIS maturation are coordinated with the expression of Kv1 and Kv2 channels, in terms of protein tethering mechanisms in this axonal domain, and to identify the functional consequences of AIS maturation on action potential generation and the regulation of neuronal excitability.

### Kv Channel Distribution in the AIS

While the uneven distribution of different Kv channel types is required for proper neuronal function, the specific cellular and subcellular distribution of distinct Kv channel proteins has not been fully elucidated [73], [74]. We observed a distinct distribution for Kv1, Kv2 and Kv7 channels in cultured hippocampal neurons. The Kv7 or KCNQ channel is expressed in the AIS of different neuronal types, including the rodent adult hippocampus and cultured hippocampal neurons [23], [46], [47], [48], [49], [50], [51]. We found that during the first three weeks of *in vitro* development, the Kv7.2 subunit was homogeneously distributed throughout the length of the AIS in the vast majority of hippocampal neurons. This is consistent with the homogeneous distribution of ankyrin G throughout the AIS, as the ankyrin G binding domains of Kv7.2 and Kv7.3 are required for their localization to the AIS [46], [48], [49].

In contrast to Kv7 channels, the Kv1.2 subunit of Kv1 channels was restricted to the distal region of the AIS. AIS compartmentalization, is evident through the segregation of different VGSCs and the enrichment of the distal AIS with Kv1 channels, and it has been linked with the specialization of the distal and proximal AIS regions in generating and back-propagating APs, respectively [13], [17], [18], [27], [28], [29], [30], [31], [32], [33], [75]. However, enrichment of the Kv1.2 subunit at the distal versus the proximal AIS is only observed in certain neuronal types, probably reflecting electrophysiological differences between neuronal populations. These include neocortical pyramidal cells in layer 2/3, interneurons, CA1 pyramidal neurons and retinal ganglion cells, but not other neuronal populations such as pyramidal neurons in layer V of the neocortex or in the CA3 region of the hippocampus [17], [18], [28], [35]. In our hippocampal cultures, only 30% of the neurons expressed Kv1.2 at the distal AIS at 21 DIV. This percentage may reflect the proportion of CA1 pyramidal neurons in our model, or alternatively a delay in Kv1.2 expression in the AIS of the neuronal population corresponding to the 60% of neurons that do not express Kv1.2.

Kv2 delayed rectifier channels include those comprised of Kv2.1 and Kv2.2 subunits, although they can also form heterotetramers with members of the silent Kv subfamilies (Kv5, 6, 8 and 9) [76]. Kv2 channels regulate excitability in hippocampal and cortical neurons rather than in playing a more classical role in action potential repolarization [37], [39], [40], [41], [42], [43], and they are mainly distributed in clusters of around 1.3 microns in diameter in the soma and proximal dendrites of neocortical and hippocampal neurons [38], [40], [77], [78], [79], [80], [81], [82], [83], [84]. Clusters of Kv2.1 have been associated with astrocytic contact points [85] being also coincident with membrane zones associated with subsurface reticulum cisterns, known to also contain IP_3_Rs [86], [87], [88], [89]. Kv2.1 clusters overlap with clusters rich in ryanodine receptor Ca^2+^ release channels and the luminal Ca^2+^ binding protein calsequestrin, suggesting the involvement of Kv2 channels in Ca^2+^ regulation [37], [82], [85]. We found that in addition to this somatodendritic domain, Kv2.2 also clusters in the AISs of cultured hippocampal cells, mainly in the proximal AIS. Hence, Kv2.2 subunits may contribute to the maintenance of the AP amplitude in hippocampal neurons by regulating inter-spike potential during high frequency firing, as occurs in neurons of the median nucleus of the trapezoid body [45]. The AIS contains the cisternal organelle, which is composed of stacks of smooth endoplasmic reticulum cisterns. The outermost of these elements is in apposition to the plasma membrane, and contains IP_3_R-expressing microdomains [70]. However, no spatial overlap appears to occur between Kv2.2-expressing AIS membrane clusters and IP_3_R1-containing microdomains (unpublished observations), suggesting that AIS Kv2 channels are not involved in the IP_3_R1-mediated Ca^2+^ dependent mechanisms in the AIS.

The clustering of Kv2.2 at the AIS described here is consistent with that of the Kv2.1 subunit in hippocampal neurons *in vitro*, and in neocortical neurons *in vivo* [44]. Whether Kv2.2 colocalizes with Kv2.1 in the AIS clusters remains unknown. At 21 DIV, Kv2.2 clusters were observed in the AIS of approximately 60% of cultured neurons, and a similar proportion of neocortical pyramidal neurons exhibited Kv2.2 somatodendritic immunostaining [38], [83]. Interestingly, the expression of Kv2.2 and Kv1.2 subunits at the AIS in cultured hippocampal neurons was mutually exclusive. It remains unclear whether these distinct expression patterns reflect differences in neuronal type within the mature hippocampal formation, or alternatively, a lag in *in vitro* Kv1.2 or Kv2.2 expression in a specific neuronal type.

### Mechanisms Mediating AIS Localization of Kv Channels

In recent years, several studies have described mechanisms responsible for the concentration of ion channels at different axonal subdomains, including protein-protein interactions, and have identified amino acid motifs involved in these interactions. However, the functions of the proteins that form complexes with Kv channels, and the mechanisms responsible for channel trafficking and clustering at the AIS have yet to be fully characterized [90].

In the AIS, the presence of ankyrin G and its interaction with the actin cytoskeleton through βIV spectrin is critical to concentrate functionally important molecules, such as VGSCs, the adhesion molecules neurofascin-186 and NrCAM, and Kv7 potassium channels [16], [46], [48], [49], [56], [57], [59], [60], [61], [62], [63], [91]. Accumulation of the latter occurs through direct binding of Kv7.2 and Kv7.3 subunits to ankyrin G via an ankyrin G binding domain similar to that found in VGSCs [48], [49], [91].

Proteins that form complexes with Kv1 subunits (Kv1.1, Kv1.2 and Kv1.4) include Caspr2, TAG-1 and ADAM22, and the cytoskeletal scaffold PSD 93. The localization of Kv1 in the AIS is dependent on the presence of PSD93/Chapsyn-110 and on PDZ domain interactions [24], [92], [93], although additional mechanisms may also be involved [74], [94]. We found that the accumulation of Kv1.2 subunits in the AIS of cultured hippocampal cells was unaffected by disruption of the actin cytoskeleton and was dependent on the presence of ankyrin G at the AIS, as these subunits were absent in neurons nucleofected with ankyrin G shRNA. While no evidence for a direct interaction between ankyrin G and Kv1.2 has been reported [20], our data indicate that the presence of ankyrin G is necessary for proper AIS development, and for the acquisition and/or maintenance of Kv1.2 and Kv2.2 expression at the AIS. This view is in line with previous studies in which ankyrin G knockdown resulted in the loss of ankyrin G-interacting proteins, such as Na^+^ channels, βIV spectrin and neurofascin-186 [20], [63], as well as the disappearance of casein kinase 2α, IP_3_R1, annexin 6, synaptopodin and α-actinin immunostaining at the AIS [69], [70], [72].

The diverse mechanisms involved in Kv2 channel clustering, including that which occurs at the AIS, remain to be fully elucidated [20], [82]. Kv2.1 subunit clusters are dynamic structures, the maintenance and localization of which may depend on the presence of a targeting motif known as the proximal restriction and clustering (PRC) signal [81], or on their interaction with scaffold proteins [95]. Kv2.1 clustering and the voltage-dependence of channel activation are also regulated by phosphorylation in response to both neuronal activity-induced Ca^2+^ influx and Ca^2+^ release from internal stores [37], [96], [97], [98]. Within clusters, Kv2.1 channels are mobile, although their mean diffusion coefficient is lower than that outside the clusters, suggesting that the clusters are corralled by part of the cortical cytoskeleton [97], [99]. Kv2.1 clusters are reported to favor cell surface regions not associated with phalloidin-positive F-actin, suggesting that they form within depressions in the cortical cytoskeleton corralled by a high density of cortical actin filaments [99], [100]. Accordingly, actin depolymerization in HEK cells and hippocampal neurons has been reported to either increase Kv2.1 cluster size [99] or induce complete cluster dissolution [97]. Kv2.1 subunit clusters in the AIS are more stable than those found in the soma [44]. Moreover, although no measurements of cluster size were performed, we found that Kv2.2 subunit clusters in the AIS were not affected by disrupting the actin cytoskeleton with cytochalasin D, which disrupts the diffusion barrier of the AIS, and deregulates the normal mobility and asymmetric distribution of other AIS proteins and lipids [64], [65], [101]. Together with the absence of Kv2.2 clusters from the AISs of ankyrin G shRNA-nucleofected neurons, these findings suggest that in addition to the actin cytoskeleton, other as yet uncharacterized molecular interactions might contribute to the stabilization Kv2 channel clusters in the AIS.
